# PCBP2 promotes immune evasion via cGAS-STING pathway in biochemical recurrence of prostate cancer

**DOI:** 10.1063/5.0250173

**Published:** 2025-03-05

**Authors:** Zeng Zhou, Tiewen Li, Yichen Zhang, Xuehao Zhou, Xiaodong Song, Shiyu Ji, Yishu Huang, Yu Zhang, Yuan Ruan

**Affiliations:** 1 Department of Urology, Shanghai General Hospital, Shanghai Jiao Tong University School of Medicine, Shanghai, China; 2 Department of General Surgery, Pancreatic Disease Center, Ruijin Hospital, Shanghai Jiao Tong University School of Medicine, Shanghai, China

## Abstract

Immunotherapy resistance is a significant obstacle in the treatment of prostate cancer (PCa), primarily due to immune evasion mechanisms. This study aims to explore cancer-intrinsic immune evasion-related genes (CIERGs) in PCa and develop a predictive signature for biochemical recurrence (BCR). Bulk RNA-seq data and single-cell RNA-sequencing (scRNA-seq) were obtained from TCGA and Gene Expression Omnibus database. The scRNA-seq data analysis revealed higher immune evasion scores in tumor cells compared to normal cells. Differentially expressed genes from TCGA-PRAD and GSE70769 cohorts were intersected with 182 core immune evasion genes, followed by univariate Cox regression, identifying 48 CIERGs significantly associated with BCR. Nonnegative matrix factorization (NMF) clustering revealed two immune evasion-related PCa subtypes. A risk signature based on CIERGs was developed using LASSO regression, and a nomogram was created to predict BCR-free survival. Among the 48 identified CIERGs, poly(C)-binding protein 2 (PCBP2) emerged as a key risk factor associated with poor prognosis in PCa, and its function was validated *in vitro*. NMF clustering identified two subtypes, with the C1 subtype having a poorer prognosis. Gene Set Variation Analysis highlighted enrichment in cell cycle, extracellular matrix receptor interaction, and transforming growth factor-beta signaling pathways in the C1 subtype. A CIERGs-based risk signature, including six key genes, was developed and validated, with the nomogram showing high predictive accuracy. *In vitro* experiments showed PCBP2 promotes PCa cell proliferation, migration, and invasion by inhibiting the cyclic GMP-AMP synthase-STING pathway. The CIERGs signature provides a precise prediction of BCR, with PCBP2 emerging as a potential therapeutic target due to its inhibition of the cGAS-STING pathway in PCa.

## INTRODUCTION

Prostate cancer (PCa) is the second most prevalent malignant tumor among men and the fifth leading cause of cancer-related deaths worldwide.^1^ The clinical treatment of localized PCa primarily involves radical prostatectomy (RP) and radiotherapy. However, approximately 30%–50% of patients may develop a biochemical recurrence (BCR) within 10 years post-RP.^2^ BCR is determined by an initial undetectable Prostate Specific Antigen (PSA) level post-surgery, followed by a detectable increase in PSA, with two or more subsequent measurements or a rise above 0.1 ng/ml.^3^ It is imperative to detect BCR at an early stage in order to provide salvage therapy with a curative aim, as BCR significantly impacts survival.^4^ Nevertheless, traditional PSA measurements often fail to accurately detect BCR at low PSA levels, which requires the use of prostate-specific antigen membrane positron-emission tomography (PSMA-PET) tracers to determine whether the cancer is locally recurrent or has metastasized.^5^ However, the widespread adoption of PSMA-PET is restricted by its high cost and complexity of application, which limits its utility in routine clinical practice.^6^ Consequently, there is an immediate need for the development of an informative tool that can accurately evaluate the prognosis of PCa.

Cancer-intrinsic immune evasion allows tumor cells to evade detection and destruction by the host immune system, thereby enabling them to survive and proliferate.^7^ This process is characterized by the secretion of inhibitory cytokines, mutations in DNA repair genes, defects in interferon signaling, downregulation of tumor antigens, and suppression of immune cell infiltration.^8^ Despite the clinical efficacy of immune checkpoint blockades (ICB) that target cytotoxic T-lymphocyte-associated protein 4 and programmed cell death 1 (PD-1)/PD-1 ligand 1 (PD-L1), only a fraction of patients achieve sustained responses as a result of the complex and heterogeneous tumor microenvironment (TME).^9,10^ Although PCa is primarily classified as an immunologically cold tumor, it is infiltrated by several immune cell types, including innate immune cells, adaptive immune cells, and immune-suppressive cells.^11^ The results of ICBs designed to alleviate the constraints on T cell proliferation have been discouraging thus far.^12^ As a result, ICBs remain effective for only a small proportion of advanced PCa patients.^13^ Recent research has revealed that targeting B7-H3 can inhibit tumor progression and reverse T cell and NK cell suppression in PTEN/TP53-deficient PCa, providing a novel route for therapeutic intervention.^14^

A recent study has identified 182 core cancer-intrinsic evasion genes that regulate the sensitivity or resistance to cytotoxic T lymphocytes (Table S1).^15^ Building upon this foundation, we analyzed single-cell RNA-sequencing (scRNA-seq) and bulk RNA-seq data from the Gene Expression Omnibus (GEO) and The Cancer Genome Atlas (TCGA) databases. Our findings revealed a significant elevation in immune evasion in PCa patients. Moreover, we explored the role of cancer-intrinsic immune evasion-related genes (CIERGs) in PCa and their association with clinical outcomes. Through univariate Cox regression analysis on data from TCGA and GEO databases, as well as 182 core cancer-intrinsic evasion genes, we identified a subset of these CIERGs that were significantly associated with patient prognosis. We distinguished two subgroups, C1 and C2, through non-negative matrix factorization (NMF) clustering. Our results indicate that poly(C)-binding protein 2 (PCBP2) influences the prognosis of PCa by suppressing the cGAS-STING pathway, which potentially upregulates inhibitory immune checkpoints such as PD-L1, and by promoting cancer cell mitosis. This study provides novel insights into the early prediction of BCR in PCa patients, highlighting the role of tumor immune evasion and its regulatory mechanisms.

## RESULTS

### Identification of CIERGs in PCa using scRNA-seq dataset

**FIG. 1.**
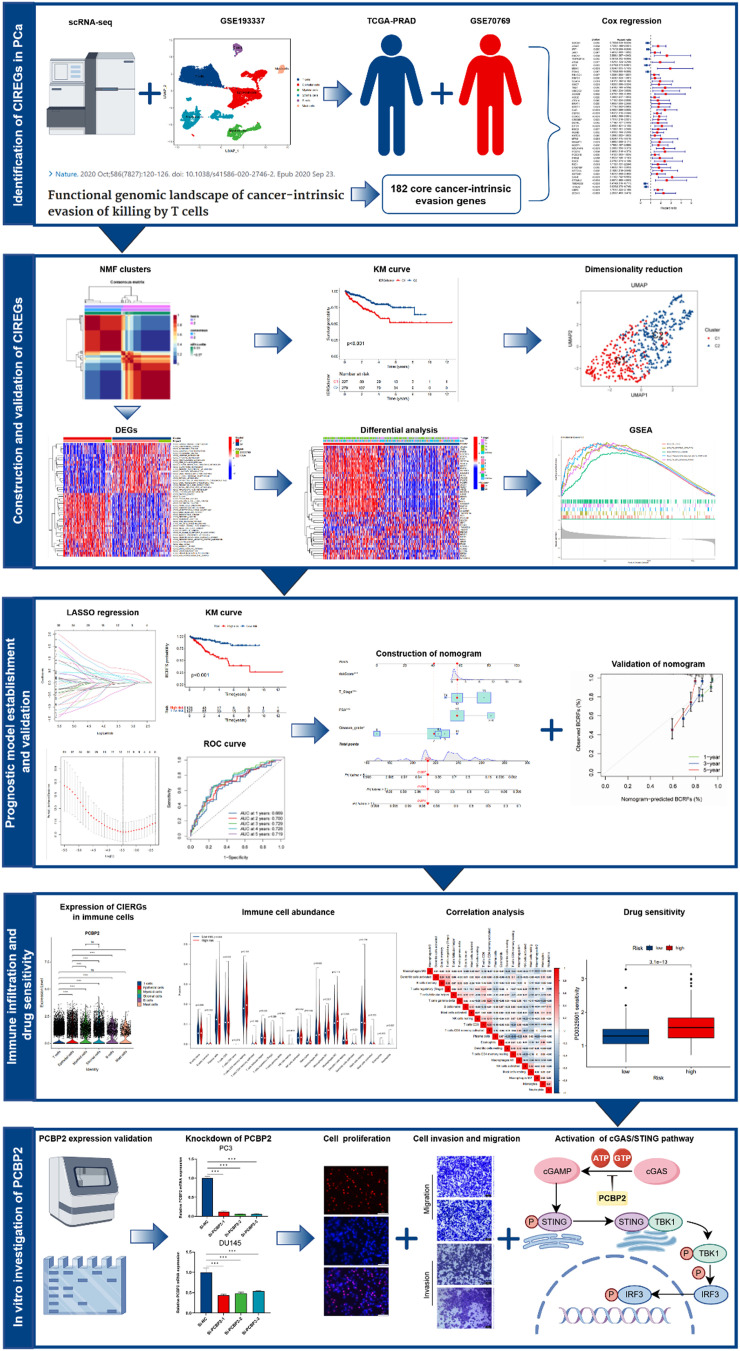
Flowchart of the CIERGs signature for predicting prognosis and immunotherapy response in PCa patients, created by Figdraw.

Figure 1 depicts the flow chart of the entire study. The GSE193337 scRNA-seq data analysis in this study identified six cell types in PCa samples. After initial processing and normalization, a total of 17 970 cells were classified into major cell types including T cells, epithelial cells, myeloid cells, stromal cells, B cells, and mast cells, as visualized using UMAP [Fig. S1(a)].^15^ Figure S1(b) shows that tumor cells exhibit significantly higher immune evasion scores compared to normal cells. Through differential analysis of the TCGA-PRAD and GSE70769 datasets, intersecting with the 182 core cancer-intrinsic evasion genes identified previously, we conducted a COX regression analysis and identified 48 CIERGs [Fig. S1(c)]. The analysis of copy number variation (CNV) rates for the identified CIERGs is presented in Fig. S1(d). Additionally, Fig. S1(e) depicts the chromosomal locations of these CIERGs.

### NMF clustering analysis of TCGA-PRAD patients

**FIG. 2.**
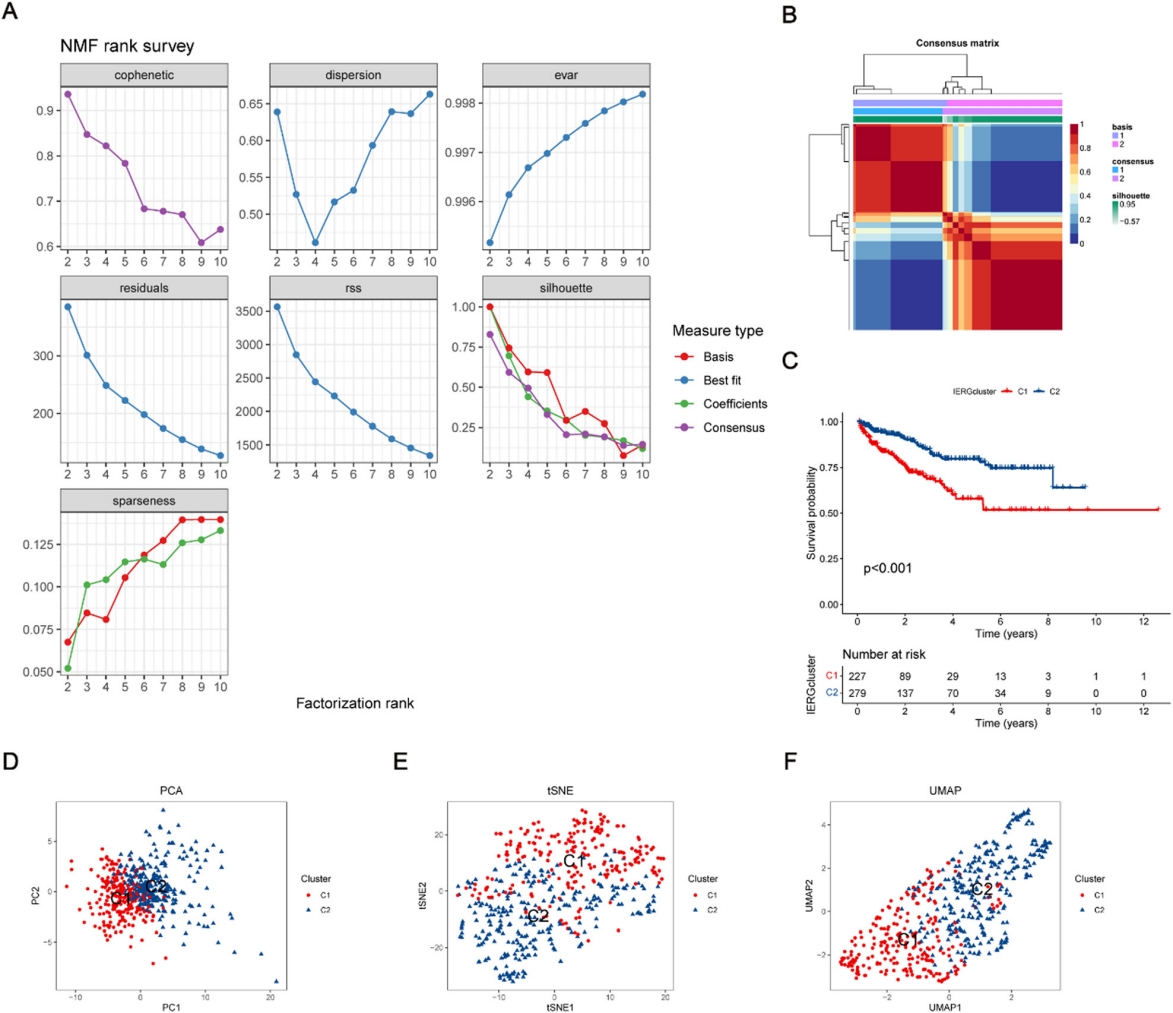
Identification of CIERGs clusters using the NMF method. (a) NMF rank survey showing rank and cophenetic correlation coefficients; (b) a consensus matrix of NMF clusters; (c) KM curve for the two clusters based on CIERGs; (d) PCA plot of the two clusters; (e) t-SNE plot of the two clusters; and (f) UMAP plot of the two clusters.

Next, an NMF consensus clustering analysis was conducted using the expression profiles of the identified CIERGs. As depicted in Figs. 2(a) and 2(b), the cophenetic coefficient and other evaluation metrics were used to determine the optimal number of clusters for the TCGA-PRAD and GSE70769 cohort, which was ultimately divided into two. Subsequently, KM survival analysis revealed a significant prognostic disparity between the clusters, with patients in the C1 cluster exhibiting a shorter median time to BCR compared to those in the C2 cluster [p < 0.001, Fig. 2(c)]. Furthermore, dimensionality reduction techniques, including PCA, t-SNE, and UMAP, unequivocally demonstrated significant discrepancies between the C1 and C2 subtypes, further validating the clustering results [Figs. 2(d)–2(f)].

### Clinical and molecular characteristics of CIERGs subtypes in PCa patients

**FIG. 3.**
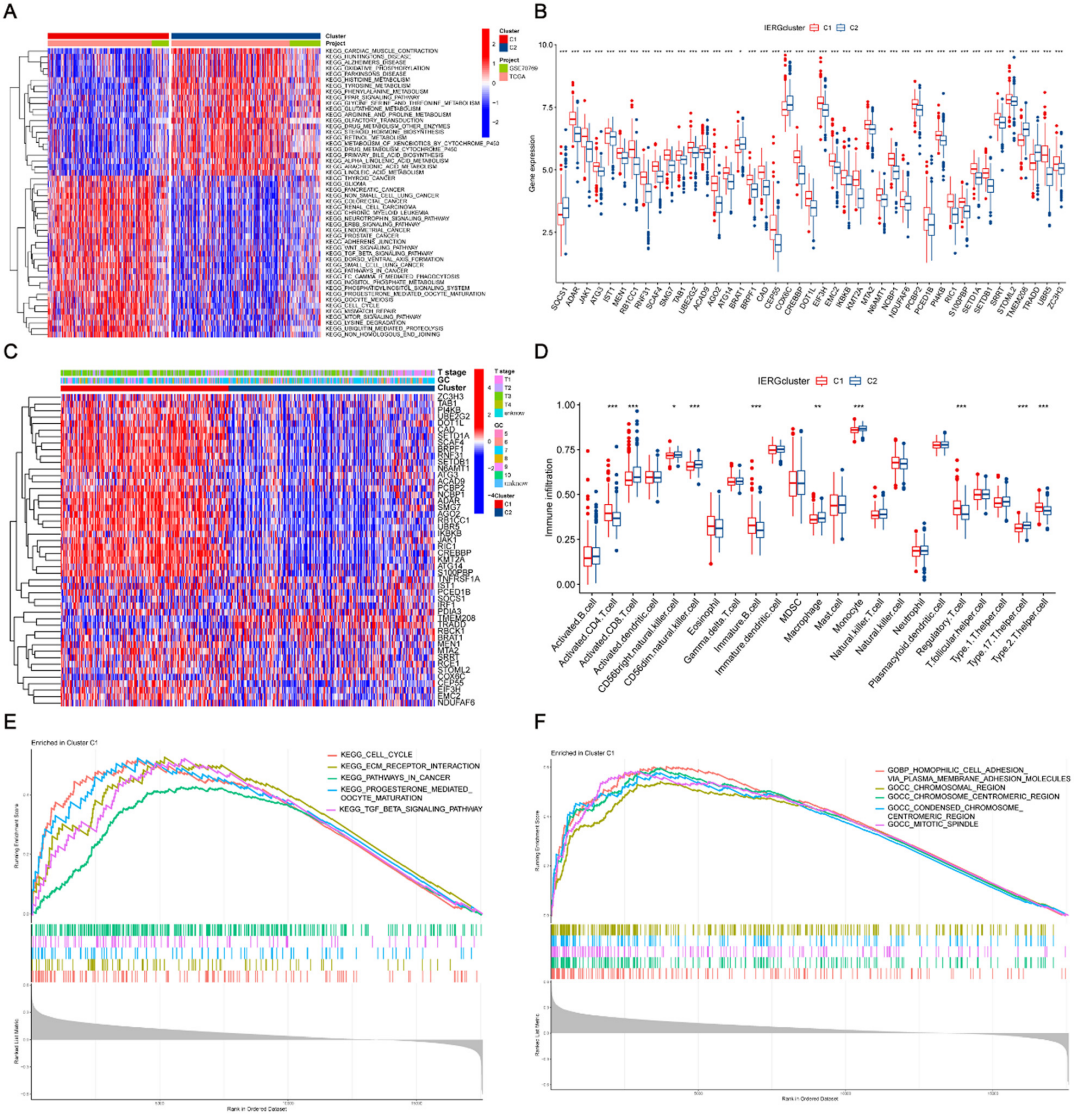
Comparative analysis of CIERGs clusters. (a) Heatmap showing DEGs between the subtypes identified through NMF clustering; (b) Box plots comparing gene expression levels of 43 prognosis-associated CIERGs across the subtypes; (c) Heatmap illustrating the relationship between clinical characteristics, CIERGs clusters, and gene expression. (d) Differential analysis of immune cell infiltration across 23 cell types between the two clusters. (e) and (f) Gene set enrichment analysis (GSEA) showing KEGG and GO pathways enriched in cluster 1.

Gene differential expression analysis was performed between the C1 and C2 subtypes, and significant differentially expressed genes (DEGs) are presented in Fig. 3(a). The expression patterns of the CIERGs were then examined in both subtypes. Consistent with our previous analyses, genes with higher expression levels in the C2 subtype were primarily associated with a better prognosis, whereas those with higher expression levels in the C1 subtype correlated with poorer outcomes [Fig. 3(b)]. The distribution of clinical features across the C1 and C2 subtypes depicts the differences in various clinical parameters between the two clusters [Fig. 3(c)]. The C1 group demonstrates a higher prevalence of high-level Gleason grades and advanced T stages compared to the C2 group. Additionally, we employed the ssGSEA method to assess the proportions of 23 tumor-infiltrating immune cells in the C1 and C2 subtypes [Fig. 3(d)]. Moreover, GO and KEGG enrichment analyses were conducted to elucidate the pathways and biological functions associated with the DEGs between the C1 and C2 subtypes. The KEGG analysis revealed that the C1 subtype was significantly enriched in pathways related to the cell cycle, extracellular matrix (ECM) receptor interaction, pathways in cancer, progesterone-mediated oocyte maturation, and the TGF-beta signaling pathway [Fig. 3(e)]. As indicated by the GO analysis, the C1 subtype was significantly enriched in processes such as homophilic cell adhesion via plasma membrane adhesion molecules, chromosomal region, condensed chromosomal centromeric region, and mitotic spindle [Fig. 3(f)].

### Establishment and validation of prognostic signatures based on CIERGs

**FIG. 4.**
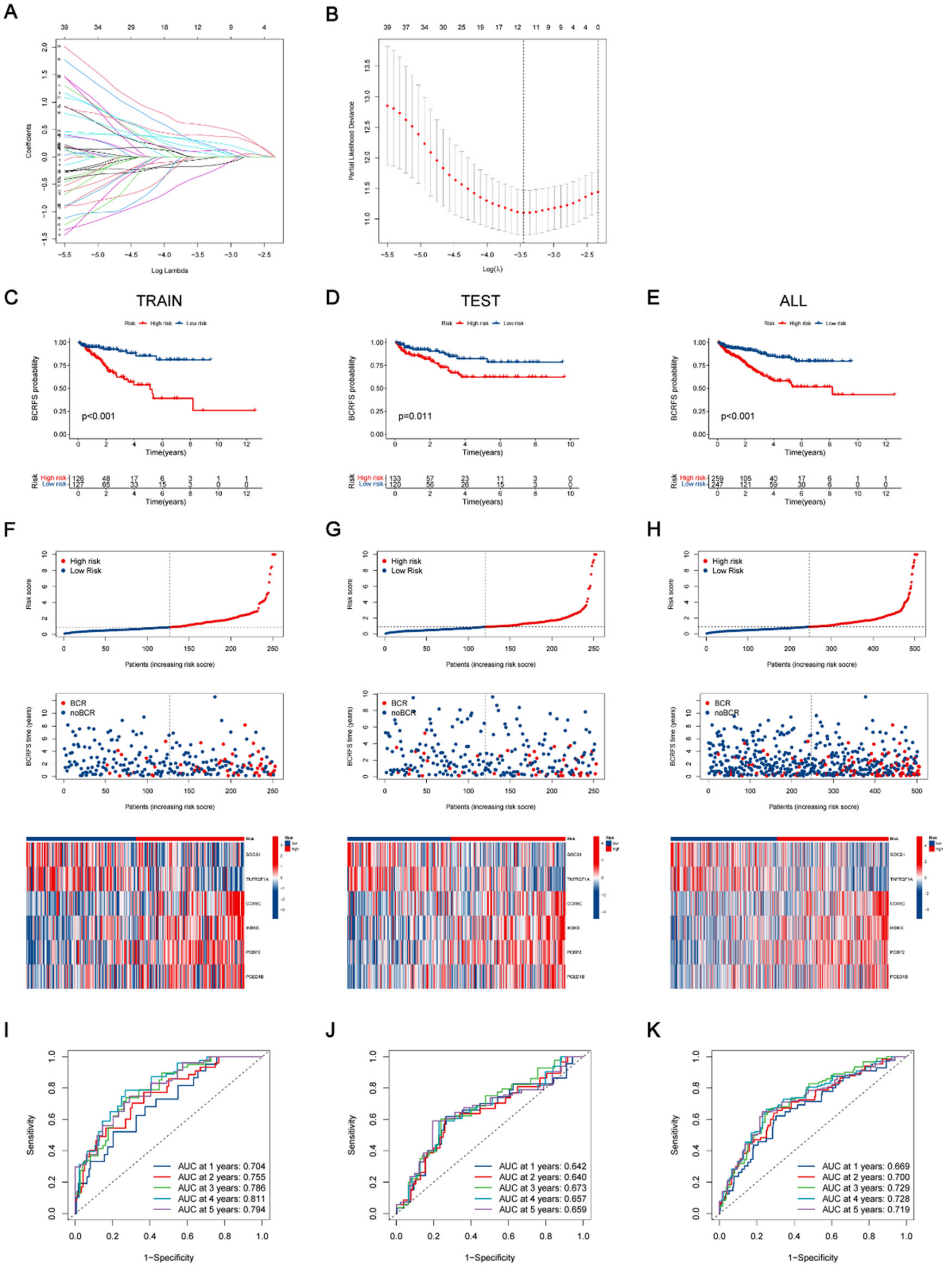
Prognostic model establishment and validation based on CIERGs. (a) The coefficients were generated by LASSO regression analysis. (b) Lambda selection in the LASSO model using tenfold cross-validation. KM curves for the training set (c), the validation set (d), and the entire dataset (e). The risk score distribution, BCRFS status, and gene expression of six CIERGs in the training set (f), the validation set (g), and the entire dataset (h). ROC curves for predicting the risk of BCR at 1, 3, and 5 years for the training set (i), the validation set (j), and the entire dataset (k).

A CIERGs based prognostic signature was developed using LASSO regression, identifying six prognostic genes: SOCS1, TNFRSF1A, COX6C, IKBKB, PCED1B, and PCBP2, which were integrated into the prognostic model. LASSO regression analysis with tenfold cross-validation was employed [Figs. 4(a) and 4(b)]. Six prognostic genes were identified and integrated into the development of the prognostic model. Patients were then classified into high- and low-risk groups based on the median signature risk score. The KM curves demonstrated a significant disparity in prognosis between these groups, with high-risk patients exhibiting substantially worse outcomes [p < 0.001, Figs. 4(c)–4(e)]. Notably, higher risk scores were strongly associated with an increased likelihood of BCR in PCa patients. Heatmaps further demonstrated the model's reliability and robustness by visualizing the distribution of risk scores, BCR status, and gene expression levels [Figs. 4(f)–4(h)]. Additionally, time-dependent ROC curves for predicting 1-, 3-, and 5-year BCRFS indicated high predictive accuracy across all datasets [Fig. 4(i)–4(k)].

### Identification of independent prognostic factors and construction of a nomogram model

**FIG. 5.**
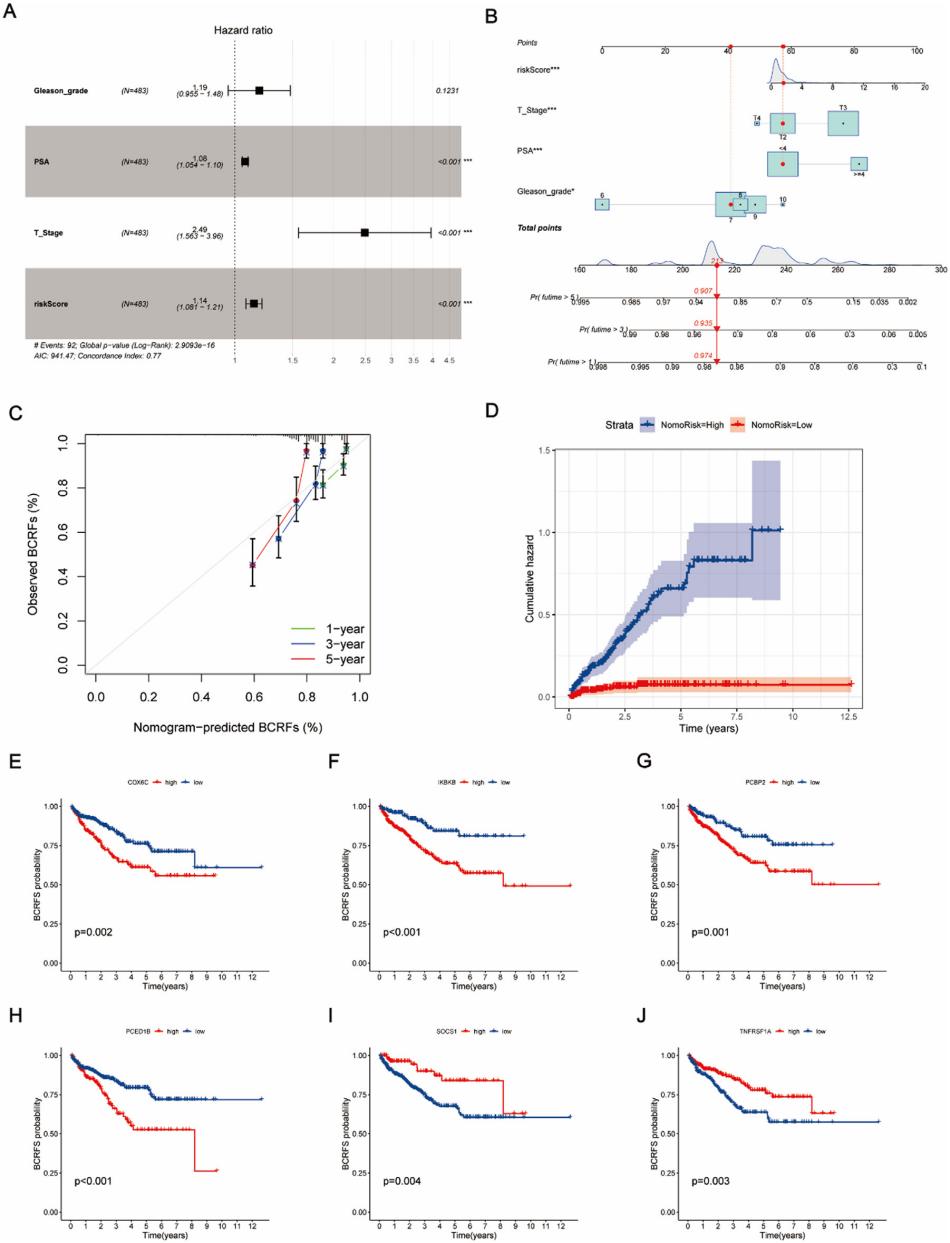
Construction and validation of a nomogram for prognostic prediction. (a) Multivariate analysis for the entire dataset. (b) Nomogram for predicting 1-, 3-, and 5-year BCRFS time based on risk score and clinical features. (c) Calibration curves illustrating the nomogram's accuracy in predicting 1-, 3-, and 5-year BCRFS in PCa patients. (d) Cumulative hazard plot for high-risk and low-risk groups based on the nomogram. (e)–(j) KM curves for BCRFS based on the expression of CIERGs.

To identify independent prognostic factors and construct a robust nomogram model that is capable of accurately predicting patient outcomes, both univariate and multivariate Cox regression analyses were conducted. The analyses included clinical variables such as Gleason grade, PSA level, T stage, and risk score. The risk score was found to be an independent prognostic factor for BCR in PCa patients [Fig. 5(a)]. After identifying these significant prognostic factors, a nomogram incorporating Gleason grade, PSA, T stage, and risk score was constructed to predict the probabilities of 1-, 3-, and 5-year BCRFS [Fig. 5(b)]. Calibration curve showed strong agreement between predicted and observed BCRFS [Fig. 5(c)]. The cumulative hazard plot differentiates between high- and low-risk groups based on the nomogram, demonstrating a significantly higher cumulative hazard for the high-risk group over 10 years [Fig. 5(d)]. Furthermore, the KM curves for BCRFS based on the expression levels of six prognostic CIERGs (COX6C, IKBKB, PCBP2, PCED1B, SOCS1, and TNFRSF1A) are shown in Figs. 5(e)–5(j).

### Analysis of six prognostic CIERGs expression and distribution in PCa

To elucidate the role of the six prognostic CIERGs within the TME of PCa, we performed a comprehensive analysis of their expression levels across various immune cell types using bulk RNA and single-cell RNA sequencing data. Figure S2(a) presents the expression levels of these genes across different cell identities, including T cells, epithelial cells, myeloid cells, stromal cells, B cells, and mast cells. Next, we visualized the tissue distribution of CIERGs within the TME using UMAP plots [Fig. S2(b)]. A comparative analysis between normal and tumor tissues reveals significant upregulation of CIERGs, such as PCBP2 in tumor-associated immune cells [Fig. S2(c)]. Additionally, the proportion of epithelial cells that express high levels of PCBP2 is significantly higher in tumor tissues compared to normal tissues [Fig. S2(d)]. Finally, the tissue distribution of epithelial cells with high and low expression of PCBP2 in normal and tumor tissues is compared using a UMAP plot [Fig. S2(e)].

#### Immunotherapy response prediction based on CIERGs

**FIG. 6.**
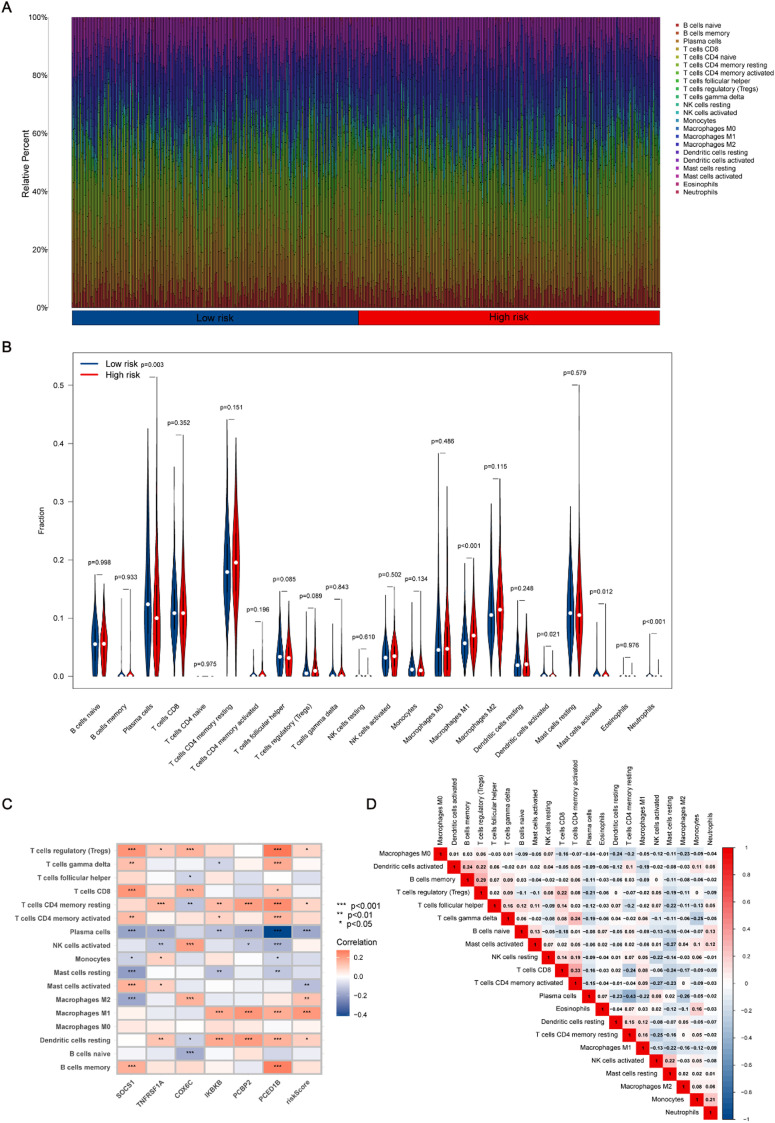
Differential analysis of immune cell infiltration between low- and high-risk subgroups. (a) Immune cell abundance differences between low- and high-risk groups. (b) Violin plots illustrating the differences in immune cell abundance between low- and high-risk groups. (c) Correlation between selected CIERGs and immune cell types. (d) Correlation analysis between risk scores and immune cell types.

We conducted an analysis of the relative percentages of various immune cell types in the low- and high-risk groups to assess the predictive capacity of the CIERGs for immunotherapy responsiveness, utilizing data from the TCGA-PRAD and GSE193337 cohorts [Fig. 6(a)]. Comparative analysis indicated that plasma cells were significantly more abundant in the low-risk group, while macrophages M1 were notably more prevalent in the high-risk group [Fig. 6(b)]. These findings were further supported by correlation analysis, which demonstrated a negative correlation between plasma cells and CIERGs expression and a positive correlation between CIERGs expression and macrophages M1 [Fig. 6(c)]. Furthermore, relationships between various immune cells highlighted the complexity and interconnectedness of the tumor immune landscape in PCa [Fig. 6(d)]. In addition, further analysis of CIERGs expression levels in relation to risk scores and clinical outcomes demonstrated that higher risk scores are associated with elevated levels of specific immune cells and functions, such as M1 and M2 macrophages [Figs. S4(a)–S4(g)]. Moreover, significant differences in the TME scores were observed between the low- and high-risk groups [Fig. S4(h)]. Specifically, the high-risk group exhibited higher stromal scores, immune scores, and ESTIMATE scores compared to the low-risk group, indicating a more complex and enriched TME. Finally, an analysis of immune cell infiltration differences between low- and high-risk subgroups in both cohorts revealed higher abundances of plasma cells, activated dendritic cells, and Type II IFN response in the low-risk group [Fig. S4(i)].

#### Chemotherapy sensitivity prediction

To investigate the chemotherapy response differences between the low- and high-risk subgroups, we evaluated the effectiveness of the risk model in predicting chemotherapy sensitivity. The analysis indicated that the high-risk group was more sensitive to several chemotherapy drugs, such as selumetinib and JAK1_8709 [Figs. S3(a) and S3(b)]. In contrast, the low-risk group showed heightened sensitivity to drugs, including AZD5991 and MM1 [Figs. S3(c) and S3(d)].

### Validation of PCBP2 expression in PCa cell lines

**FIG. 7.**
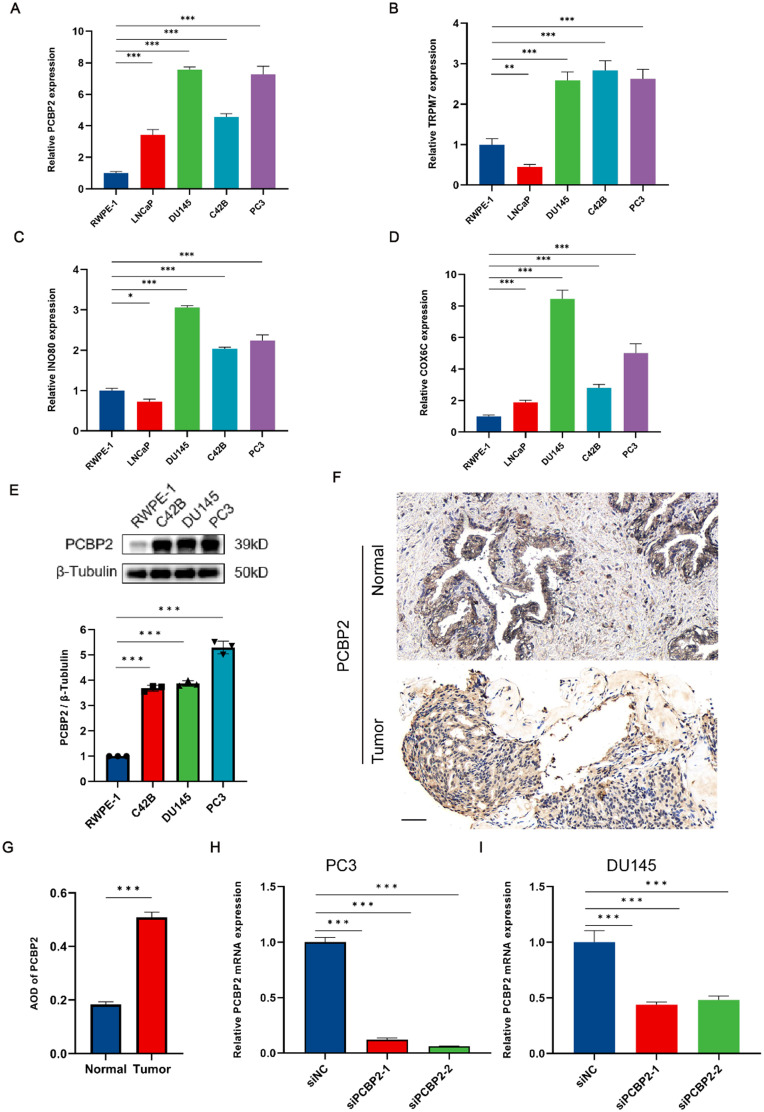
*In vitro* validation of CIERGs expression levels. (a)–(d) The relative expression levels of PCBP2, TNFRSF1A, NDRG1, and COX6C in various prostate cell lines (RWPE-1, C4-2B, DU145, and PC3). (e) Western blot analysis confirming protein levels of PCBP2 across prostate cell lines. (f) and (g) IHC staining illustrating PCBP2 expression in normal and tumor prostate tissues (scale bar, 50 *μ*m). (h) and (i) The knockdown efficiency of PCBP2 in PC3 and DU145 cells. *** Means P < 0.001.

Based on previous analyses identifying TRPM7, INO80, COX6C, and PCBP2 as risk genes, we conducted validation studies on these CIERGs in PCa cell lines. Initially, quantitative reverse transcription polymerase chain reaction (qRT-PCR) analysis revealed that the mRNA levels of these CIERGs were significantly higher in PCa cell lines compared to the normal prostate cell line RWPE-1 [Figs. 7(a)–7(d)]. Notably, PCBP2 expression was consistently elevated across all PCa cell lines. Based on the immunohistochemistry (IHC) data from the HPA database, PCBP2 showed significantly higher expression in tumor tissues compared to normal tissues [Fig. S5(a)]. In contrast, COX6C and TRPM7 did not show significant differences between prostate tumor and normal tissues, and INO80 lacked IHC data [Figs. S5(b) and S5(c)]. PCBP2 was chosen for additional analysis among these genes due to its significantly higher expression in tumor tissues than in normal tissues. To further analyze the expression of PCBP2 across various cancers, we examined data from the TCGA-GTEx and TCGA databases. The expression of PCBP2 was significantly higher in most tumors [Figs. S6(a) and S6(b)], which was consistent with the expression levels observed in 11 types of tumors with paired samples in the TCGA dataset [Fig. S6(c)]. Subsequently, Western blot analysis further confirmed the elevated protein expression of PCBP2 in PCa cell lines, including DU145, C42B, and PC3, compared to RWPE-1 [Fig. 7(e)]. This finding was further confirmed by IHC data obtained from PCa tissue samples from patients at Shanghai General Hospital [Figs. 7(f) and 7(g)]. For the diagnostic analysis, the ROC curves indicated that PCBP2 has high diagnostic potential across various cancers [Fig. S6(d)].To investigate the function of PCBP2 in PCa, we performed knockdown experiments using two strands of siRNA targeted at PCBP2 and validated their efficiencies by qRT-PCR [Figs. 7(h) and 7(i)].

### Effects of PCBP2 knockdown on Pca cell lines

**FIG. 8.**
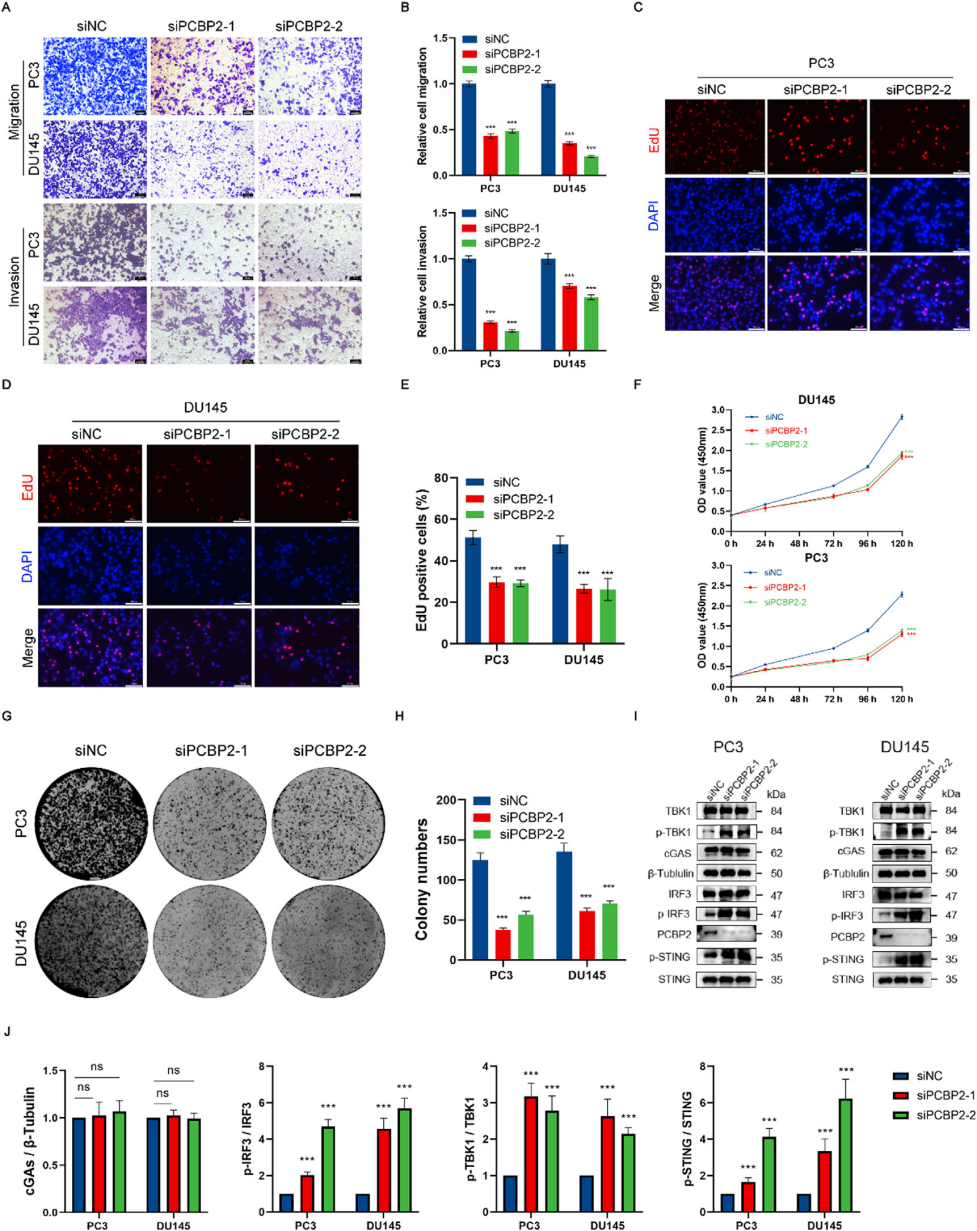
Cell function experiment of PCBP2 in PCa. (a) Transwell assays were used to detect the effects of PCBP2 knockdown on cell migration and invasion (scale bar, 100 *μ*m). (b) Quantification of cell migration and invasion after PCBP2 knockdown. Relative cell invasion is expressed as percent change (means ± SEM) compared with the controls, based on the data from three independent experiments. (c) and (d) The scratch assay was performed to evaluate cell migration in DU145 and PC3 cells transfected with either PCBP2-siRNA or NC-siRNA. Cell images were captured using an inverted microscope (magnification, ×10). EdU assays were conducted to assess the effect of PCBP2 knockdown on cell proliferation (scale bar, 100 *μ*m). (e) Quantification of EdU-positive cells showing a decrease in cell proliferation following PCBP2 knockdown. (f) CCK-8 assays were performed to evaluate the reduction in cell viability after PCBP2 knockdown. (g) and (h) Colony formation assays indicate reduced cell proliferation ability following PCBP2 knockdown. (i) and (j) Western blot analysis shows that the knockdown of PCBP2 increased the phosphorylation of STING, TBK1, and IRF3 in prostate cancer cell lines. *** Means P < 0.001.

To investigate the *in vitro* effects of PCBP2 knockdown on Pca cell lines, we employed two siRNAs to knock down PCBP2 in PC3 and DU145 cells. After PCBP2 knockdown, Transwell migration and invasion assays demonstrated a significant reduction in the migratory and invasive abilities of PC3 and DU145 cells [Figs. 8(a) and 8(b)]. Additionally, EdU incorporation assays showed a marked decrease in cell proliferation following the knockdown of PCBP2, with fewer EdU-positive cells observed in the PCBP2 knockdown groups [Figs. 8(c)–8(e)]. Furthermore, CCK-8 assays also indicated reduced cell viability after PCBP2 knockdown [Fig. 8(f)]. Colony formation assays further confirmed a significant reduction in cell proliferation ability following the knockdown of PCBP2 [Figs. 8(g) and 8(h)]. These results underscore the crucial role of PCBP2 in regulating Pca cell behavior, including migration, invasion, and proliferation. Given the significance of the cGAS-STING pathway in tumor immune evasion, we are determined to investigate whether PCBP2 influences Pca growth through this pathway.^16^ Finally, Western blot analysis confirmed the knockdown of PCBP2 and revealed increased levels of p-TBK1, p-IRF3, and p-STING, indicating the activation of the cGAS-STING pathway, which is consistent with the findings of Gu and coworkers [Figs. 8(i) and 8(j)].^17^

#### PCBP2 regulates prostate cancer cell lines via the cGAS-STING pathway

**FIG. 9.**
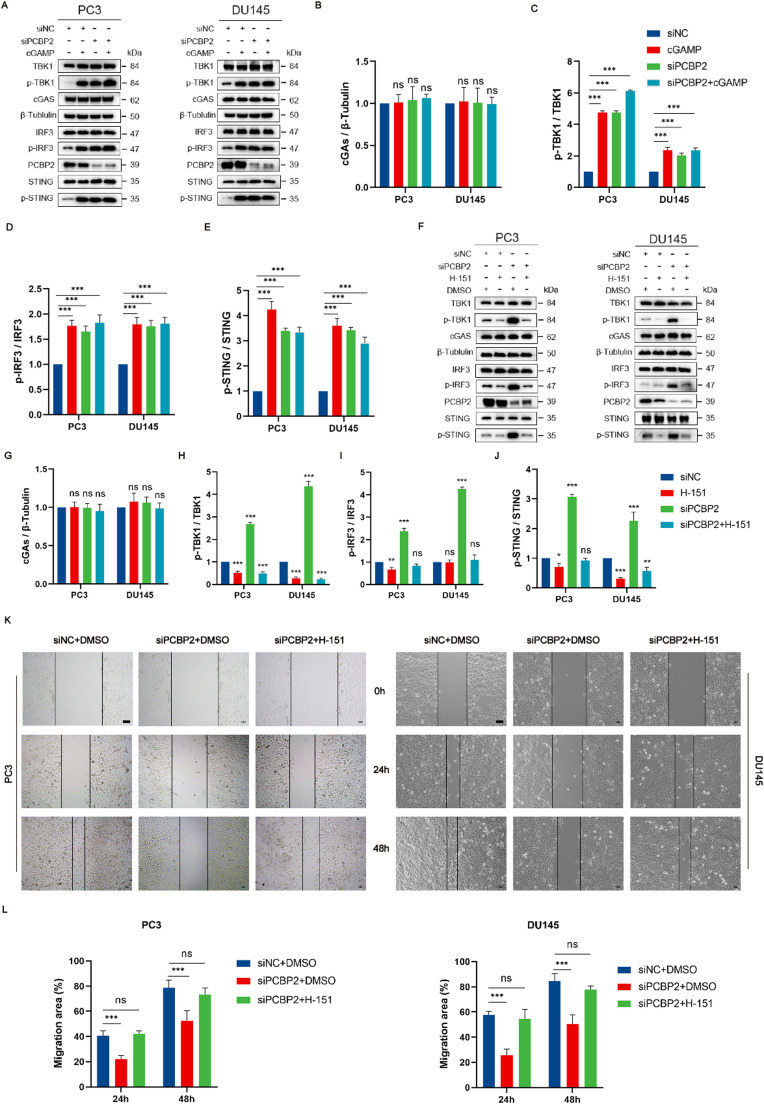
PCBP2 knockdown activates the cGAS-STING pathway and suppresses PCa cell migration. (a)–(e) Western blot analyses indicate that PCBP2 knockdown or cGAMP treatment markedly elevates phosphorylation of STING, TBK1, and IRF3 in PC3 and DU145 cells. (f)–(j) The STING inhibitor H-151 significantly reduces these phosphorylation levels induced by siPCBP2. (k) and (l) Scratch assays show that siPCBP2 impairs cell migration, whereas H-151 partially reverses this effect (scale bar, 100 *μ*m). ***Means p < 0.001. DMSO, dimethyl sulfoxide.

We identified a regulatory role for PCBP2 in the cGAS-STING pathway in PCa cell lines. Therefore, we postulated that knockdown of PCBP2 inhibits PCa cell migration by activating the STING pathway. To investigate this, PC3 and DU145 cells transfected with siNC or siPCBP2 were subsequently incubated with the STING agonist cGAMP (3 *μ*M) for 12 h, followed by Western blot analyses. The results showed that the levels of p-TBK1, p-IRF3, and p-STING were markedly elevated in the siPCBP2 group, similar to the effect observed under cGAMP treatment [Figs. 9(a)–9(e)], suggesting that PCBP2 knockdown promotes cGAS-STING pathway activation.

Subsequently, siPCBP2-transfected cells were treated with the STING inhibitor H-151 (5 *μ*M) for 48 h. H-151 covalently binds to STING at Cys91 and undergoes intramolecular rearrangement.^18^ Western blot data demonstrated that the elevated levels of p-TBK1, p-IRF3, and p-STING induced by siPCBP2 were substantially reduced by H-151 [Figs. 9(f)–9(j)], confirming that these effects rely on STING activation. Furthermore, scratch assays indicated that siPCBP2 significantly impaired the migratory capacity of PC3 and DU145 cells, while this effect was partially reversed by H-151 [Figs. 9(k) and 9(l)]. Altogether, these findings indicate that PCBP2 knockdown suppresses PCa cell migration by activating the cGAS-STING pathway, and that inhibition of STING can partially mitigate this effect.

## DISCUSSION

PCa has emerged as a primary health concern among men worldwide, with significant global incidence variations.^19^ BCR in PCa is influenced by various factors, including tumor stage, Gleason score, preoperative PSA levels, and nutritional status.^20^ Van den Broeck *et al.* found that BCR correlated with poorer survival, possibly indicating the presence of minute residual cancer cells or metastatic foci.^4^ Therefore, a more precise predictive system can enhance risk assessment and treatment strategies for PCa patients.

Cancer-intrinsic immune evasion could occur through the development of defects or insufficiencies in antigen presentation.^21^ Despite the strategic approach proposed by Beatty and Gladney for effective cancer immune surveillance, this strategy does not consistently produce effective therapeutic outcomes across all tumor types.^22^ Thus far, the PD-1 antibody has not demonstrated a significant benefit in PCa patients, which suggests that the level of PD-1 expression may not be the most reliable predictor of survival time.^23^ Additionally, the efficacy of novel immune checkpoints for the treatment of PCa, including B7-H3, has been demonstrated.^14^ Hence, it is imperative to conduct additional research to create more effective immunotherapy strategies for PCa. In this study, we initially observed that an elevated BCR rate among PCa patients was associated with severe immune escape by analyzing scRNA-seq data. After employing the NMF algorithm for patient clustering, we observed that patients in C1 had a poorer prognosis. Furthermore, we established a signature for BCR-associated CIERGs to determine the risk scores of patients. Additionally, we developed and validated a nomogram based on the risk scores and other clinical characteristics for predicting BCR and immunotherapeutic responsiveness in PCa patients.

We identified 48 CIERGs that were significantly correlated with BCR in PCa patients through univariate Cox regression analysis of the TCGA-PRAD and GSE70769 datasets, intersected with the 182 core cancer-intrinsic evasion genes. Based on KEGG pathways analysis, we found that the C1 subtype was mainly enriched in cell cycle, ECM receptor interaction, TGF-beta signaling pathway, pathways in cancer, and progesterone mediated oocyte maturation. The analysis of Gene Ontology for Cellular Component (GOCC) revealed a substantial enrichment in biological processes that involve cell adhesion, as well as components such as the mitotic spindle and condensed chromosome centromeric regions. Transforming growth factor-beta (TGF-β), a key immune evasion mechanism in various cancers, is produced by several cell types within the immune system and the TME.^24^ Regardless of the TGF-β produced by the tumor, Donkor *et al.* found that T cell-generated TGF-β1 is a fundamental requirement for tumors to evade immune surveillance using an endogenous genetic model for PCa.^25^ These observations indicate that TGF-β signaling may hinder the priming of tumor antigen-specific T cells.

Through LASSO regression analysis, we established a BCR-associated CIERGs signature, including SOCS1, TNFRSF1A, COX6C, IKBKB, PCED1B, and PCBP2. SOCS1 inhibits the autophosphorylation site of JAK2, reducing STAT3 activation and ultimately impeding the progression of the cell cycle in PCa cells.^26^ Jung *et al.* demonstrated that TNFRSF1A, by interacting with HMGB1, significantly activates the NF-κB signaling pathway, thereby facilitating the progression and metastasis of PCa.^27^ As a critical component of the mitochondrial inner respiratory chain, the heightened expression of COX6C is essential for the proliferation of malignant tumors.^28^ Wang *et al.* demonstrated that COX6C is associated with poor prognosis in prostate cancer.^29^ Liang *et al.* suggested that the upregulation of IKBKB is crucial for the development of enzalutamide resistance in castrate-resistant PCa.^30^ At the mRNA level, PCBP2 expression is consistently elevated across all representative prostate cancer stages and cell line models, highlighting its potential significance in the immune regulation of PCa. Therefore, this CIERGs signature is a multifaceted mechanism in PCa that influences cell cycle regulation and immune response modulation, thereby offering vital insights into potential targets for innovative treatment strategies.

The poly(C)-binding protein 2 (PCBP2), which is recognized for its direct interactions with single-stranded poly (C) motifs, has been identified as a prognostic marker and potential therapeutic target in a variety of cancer types. It is responsible for the post-transcriptional and translational regulation of multiple signaling molecules.^31–33^ Gu *et al.* demonstrated that PCBP2 is instrumental in the regulation of the cyclic GMP-AMP synthase (cGAS) enzyme's activity, which in turn facilitates the proper cGAS-STING signaling transduction.^17^ cGAS acts as a cytosolic sensor that detects pathogenic DNA or damaged self-DNA.^34^ Upon binding to DNA, cGAS is activated and catalyzes cGAMP synthesis, which subsequently binds to STING to activate downstream signaling pathways, thereby inducing the production of IFNs and inflammatory cytokines.^35^ In hepatocellular carcinoma, activation of the cGAS-STING pathway has been shown to upregulate PD-L1 expression via the STING-TBK1-IRF3 axis, promoting immune evasion.^36^ Similarly, our findings suggest that PCBP2 may suppress the cGAS-STING pathway in prostate cancer, potentially leading to PD-L1-mediated immune evasion. Ma *et al.* discovered that docetaxel activates the antitumor immune response and promotes T cell infiltration through the cGAS-STING pathway, suggesting that cGAS-STING pathway may be a promising target for future immunotherapy in PCa.^37^ The regulatory actions of PCBP2 on critical signaling pathways, including TGF-β/Smad, are of particular interest owing to their potential impact on the growth of gliomas.^38^ Curcusone C, a promising anti-PCa agent, targets PCBP2 in the context of PCa, potentially affecting this pathway and the apoptotic balance of Bax/Bcl-2.^32^ These results emphasize the importance of additional research to gain a more comprehensive understanding of the impact of PCBP2, particularly in the immune evasion of PCa. Our findings indicate that PCBP2 may be a viable target for the regulation of ECM remodeling and the enhancement of immunotherapy responsiveness in PCa.

**FIG. 10.**
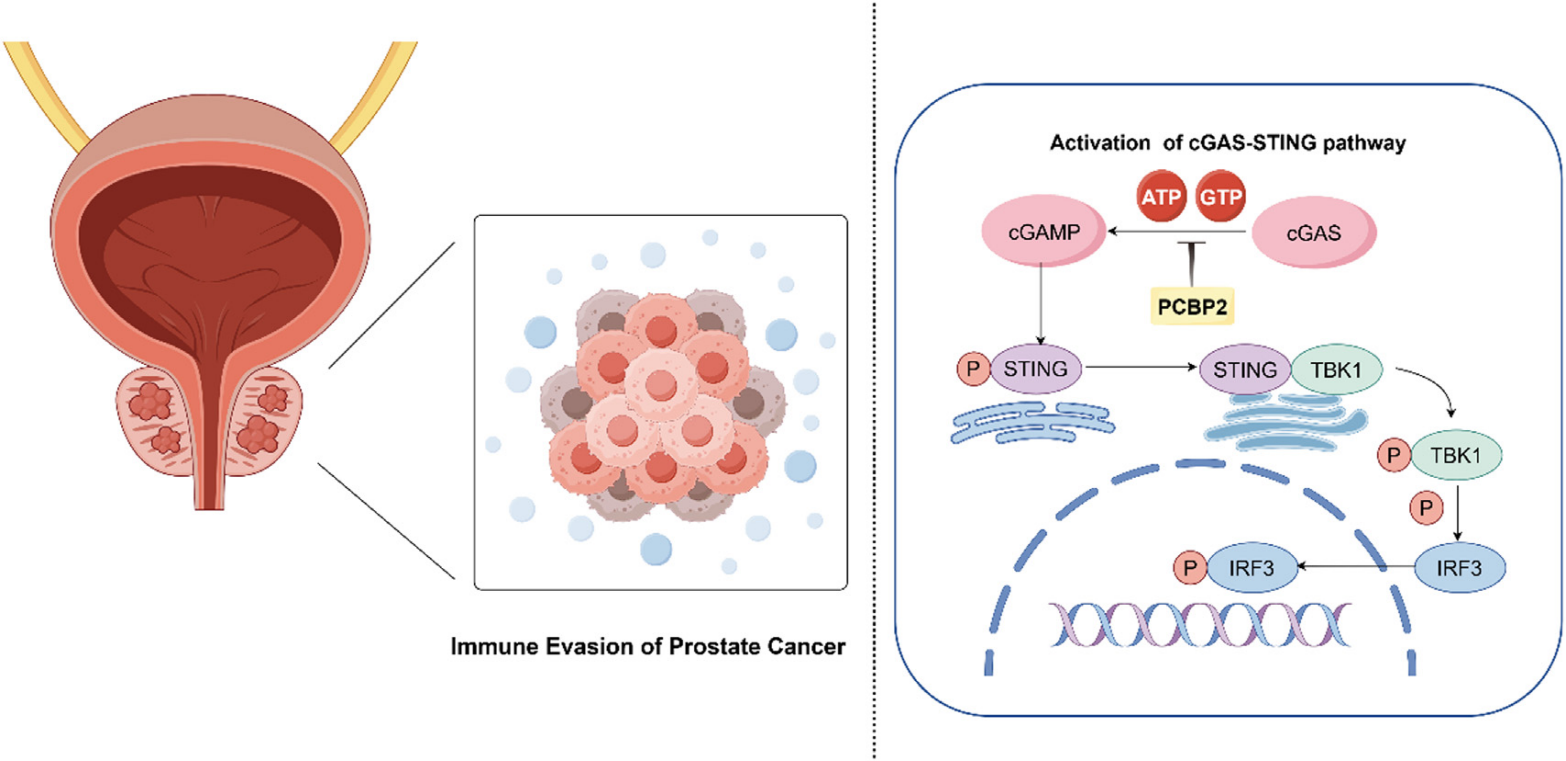
The diagram illustrates the role of PCBP2 in immune evasion in prostate cancer. PCBP2 inhibits the enzymatic activity of cGAS, reducing the conversion of ATP and GTP into cGAMP. The activation of the downstream cGAS-STING signaling pathway is suppressed, leading to decreased phosphorylation and activation of STING, TBK1, and IRF3. This results in the attenuation of the immune response against prostate cancer cells.

While the cGAS-STING pathway has been well studied in tumor immunity, our findings provide novel insights into how PCBP2 specifically suppresses this pathway in prostate cancer, leading to immune evasion. Beyond its role in cGAS inhibition, PCBP2 may also interact with other immunosuppressive mechanisms, such as the TGF-β/Smad pathway or ECM remodeling, further contributing to an immune-resistant tumor microenvironment.^38^ These findings suggest that targeting PCBP2 could not only inhibit tumor progression but also enhance immunotherapy efficacy, highlighting the need for further investigation into its broader regulatory functions in prostate cancer immunity. Figure 10 illustrates the molecular mechanism by which PCBP2 promotes PCa proliferation and invasion through the inhibition of the cGAS-STING pathway.

Despite being the first study to combine scRNAseq data from human prostate tissues with multiple bulk RNA-seq datasets for patient classification and the development of CIERGs-related collections, this study also has several limitations. First, the inherent constraints of data mining from public databases necessitate additional clinical validation. These bioinformatics insights will be translated into clinical settings in the future, with a particular emphasis on the validation of the efficacy of targeting PCBP2 in patients who have undergone RP treatment. Second, despite having examined the clinical characteristics of 506 patients, the study would benefit from a larger and more diverse patient cohort. It is imperative to increase the sample size to verify our findings and improve their generalizability, particularly in revealing the nuanced roles of PCBP2 across various PCa subtypes. Finally, the *in vitro* experiments provided preliminary validation of PCBP2's involvement in PCa. However, to fully elucidate its functions and mechanisms, particularly in the context of immune evasion, comprehensive *in vivo* studies and mechanistic investigations are required. Additionally, this study did not investigate the precise targets and mechanisms by which PCBP2 affects the cGAS-STING pathway, underscoring the necessity of additional fundamental research in this field.

## CONCLUSION

In summary, this study is the first to establish a significant link between BCR and immune evasion in PCa, identifying immune evasion subtypes and creating a predictive model. We developed a signature based on six prognostic genes, leading to a nomogram that accurately predicts PCa prognosis. *In vitro* experiments highlighted PCBP2's potential as a diagnostic biomarker and immunotherapy target by inhibiting the cGAS-STING pathway, underscoring the importance of immune evasion in PCa and provides new opportunities for personalized immunotherapy.

## METHODS

### Data source

The gene expression profiles and clinical data for PCa were retrieved from the TCGA database via the TCGA Data Portal, with the dataset identified as TCGA-PRAD. The scRNA-seq data were sourced from the GEO database, under the accession number GSE193337.^39^ Patients with less than 30 days of follow-up or without BCR information were excluded after the TCGA-PRAD and GSE70769 datasets were merged and batch effects were removed using the “sva” package.^40^ The final analysis cohort consisted of 506 samples, with 416 from TCGA-PRAD and 90 from GSE70769.^41^

### scRNA-seq data analysis and classification of cancer-intrinsic immune evasion subtypes

In this study, we utilized the “Seurat” package to analyze the scRNA-seq data from the GSE193337 dataset to identify intrinsic immune evasion subtypes in cancer.^42^ Cells with gene expressions outside the 200–4000 range or high mitochondrial gene expression (pctMT > 15%) were excluded. Subsequently, the FindVariableFeatures function in Seurat was used to select 2000 highly variable genes for data normalization. Principal component analysis (PCA) was then conducted, followed by data visualization with the t-distributed stochastic neighbor embedding (t-SNE) and uniform manifold approximation and projection (UMAP) methods. Finally, cell types and subtypes were annotated and visualized based on specific marker gene expressions.

### Identification of CIERG subgroups through NMF clustering

From the TCGA-PRAD and GSE70769 cohorts, the differentially expressed genes (DEGs) were identified and intersected with the 182 core cancer-immune evasion genes. Using univariate Cox regression analysis, we identified 48 CIERGs significantly associated with BCR. Subsequently, our study applied the NMF algorithm to stratify CIERGs into 2–10 distinct expression profiles within the TCGA-PRAD dataset. This optimal cluster count was then rigorously validated through integrative analyses, leveraging the CIERGs' expression landscapes to underpin our subtype categorization. Furthermore, we investigated the prognostic implications of these subtypes in relation to BCR, utilizing Kaplan–Meier (KM) curves.

### Functional analysis and gene expression differences between CIERGs clusters

In our detailed exploration of cancer-intrinsic immune evasion mechanisms, we strategically applied the “limma” package for differential expression analysis, pinpointing significant DEGs with a stringent threshold of |log FC| > 1 and a false discovery rate less than 0.05.^43^ Furthermore, these DEGs were subjected to a comprehensive enrichment analysis and further categorized through Gene Set Variation Analysis (GSVA) within the Kyoto Encyclopedia of Genes and Genomes (KEGG) framework.^44^ The enriched pathways highlighted by these analyses were visualized through heatmaps, and a Gene Ontology (GO) enrichment analysis delineated the biological functions and processes associated with the CIERGs, providing a multidimensional view of their role in cancer's ability to evade immune detection.

### Construction and validation of the BCR-related CIERGs signature

To construct a BCR-related CIERGs signature, we employed LASSO regression analysis, facilitated by the “glmnet” package, to minimize overfitting and refine the selection of prognostic genes. Our integrated dataset was distributed equally between the training and validation sets, each of which contained 253 samples. This division facilitated the empirical testing and validation of the model's robustness. Genes that were significantly associated with BCR were identified by employing established selection criteria, such as p-value thresholds and fold change. Following the model's construction, each sample was assigned a risk score, calculated using LASSO-derived coefficients and gene expression data. Samples were stratified into high- and low-risk groups based on the median risk score from the training set.

### Nomogram construction and risk group clinical feature analysis

A nomogram to predict 1-, 3-, and 5-year biochemical recurrence-free survival (BCRFS) times based on the BCR-related CIERGs signature was constructed using the TCGA-PRAD and GSE70769 datasets. The nomogram's prognostic accuracy was validated through calibration curves compared with actual patient outcomes. Additionally, the association between clinical features and CIERGs signature was analyzed through univariate and multivariate Cox regression, identifying independent prognostic factors. The nomogram's clinical applicability and utility in predicting BCR are further demonstrated by this method, which divides patients into low- and high-risk categories.

### Analysis of immune landscapes and drug sensitivity prediction

To evaluate the immune landscape in PCa and its implications for immunotherapy response, our study harnessed TME scores to stratify patients based on their immune microenvironment. Furthermore, the Tumor Immune Dysfunction and Exclusion (TIDE) analysis offered a critical perspective that enabled us to evaluate the efficacy of immunotherapy by distinguishing between high- and low-risk groups, as identified by risk scores. This differentiation was instrumental in predicting immunotherapy outcomes, supported by chi-square tests for statistical validation. Additionally, we explored the implications of these immune profiles for drug sensitivity, employing the “oncoPredict” package to forecast responses based on the distinct immune evasion characteristics identified.

### Assessment of TME and immune evasion using advanced computational methods

This study employed advanced computational techniques, namely, Microenvironment Cell Populations-counter and Estimating the Proportion of Immune and Cancer Cells, to evaluate tumor immune evasion within the microenvironment using RNA-seq data.^45,46^ Furthermore, we utilized the ESTIMATE algorithm to calculate stromal scores in PCa patients, providing insights into the tumor-stroma interactions and microenvironmental composition.^47^

### Comprehensive analysis of pan-cancer data from TCGA

We explored bulk RNA data from a range of cancers in the TCGA database, focusing on the expression of the PCBP2. We employed “stats” and “car” packages for preprocessing and “ggplot2” for visualization, ensuring high-quality data. The Wilcoxon rank-sum test allowed for the statistical analysis of PCBP2's differential expression across cancer types. This method, aligned with the TCGA data section's methodologies, facilitated the identification of distinct PCBP2 expression patterns.

### Cell culture and transfection

Prostate epithelial cell (RWPE-1) and PCa cell lines (PC-3, DU-145, and C42B) were acquired from the Cell Bank of the Shanghai Institutes for Biological Sciences, Chinese Academy of Sciences. RWPE-1 cells were cultured in Keratinocyte Serum-Free medium, enriched with 0.05 mg/ml bovine pituitary extract and 5 ng/ml epidermal growth factor. The PCa cell lines were grown in RPMI-1640 medium supplemented with 10% fetal bovine serum and 1% penicillin/streptomycin. All cells were cultured at 37 °C in a 5% CO_2_ environment. Cell transfections were performed as described.^48^ Briefly, PCa cells at a density of 1 × 10^6^ cells/well were seeded in 6-well plates and transfected using Lipofectamine™ 3000. The siRNA sequences were as follows: siPCBP2#1 (5′-GGAGAAUCAGUUAAGAAGAUGTT-3′), siPCBP2#2 (5′-GGCUCAAUAUCUAAUCAAUGUTT-3′), and control siRNA (5′-UUCUCCGAACGUGUCACGUTT-3′). The 2′3′-cGAMP (SML1229) was purchased from Sigma and transfected into the cells at 3 *μ*M. Η151 was purchased from Sigma (SML2437).

### Colony formation assay

Cell colony formation capacity was determined with the colony formation assay. 300 cells were seeded into each well of a six-well plate and incubated for 2 weeks until obvious colonies formed. Then, the colonies were fixed using ethanol and stained with 1% crystal violet. Finally, the colonies were counted and subjected to quantitative analysis.

### Validation of gene expression by *in vitro* qRT–PCR

To confirm the expression levels of PCBP2, quantitative reverse transcription PCR (qRT-PCR) was utilized on RWPE-1 and various PCa cell lines (LNCaP, PC-3, DU-145, and C42B). Cellular RNA was isolated using the SPARKeasy cell RNA kit (*Shandong Sparkjade Biotechnology Co., Ltd*), followed by cDNA synthesis employing a reverse transcription kit (HiScript IV RT Super-Mix, *Vazyme Biotech Co., Ltd*). The SYBER Green method was then applied in real-time PCR to measure the expression of the target genes. The specific primer sequences used are as follows: PCBP2: forward primer (5′-GGGCTTGGGCAGAGAAGATAAATGG-3′) and reverse primer (5′-CACTTGGGAGGAGGGAGGCTAAG-3′); and GAPDH: forward primer (5′-AAGGTGAAGGTCGGAGTCAAC-3′) and reverse primer (5′-GGGGTCATTGATGGCAACAATA-3′).

### Western blotting

Proteins were extracted, and their expression levels were analyzed using Western blotting analysis as described previously.^49^ Briefly, cell lysates were subjected to separation using 10% sodium dodecyl sulfate-polyacrylamide gel electrophoresis (SDS-PAGE), and the proteins were transferred onto polyvinylidene difluoride (PVDF) membranes (Merck Millipore, USA). The membranes were then incubated with primary antibodies overnight at 4 °C, followed by incubation with the appropriate secondary antibodies. Target proteins were detected and quantified using grayscale analysis with the Odyssey CLx Imaging System (LI-COR, USA). The primary antibodies were applied as follows: anti-beta-tubulin (EM0103, *HUABIO*), anti-PCBP2 (15070-1-AP, *Proteintech*), anti-cGAS (HA500023, *HUABIO*), anti-STING (ET1705-68, *HUABIO*), anti-phospho-STING (p-STING, TA7416, *Abmart*), anti-TBK1(HA601044, *HUABIO*), anti-phospho-TBK1 (p-TBK1, *CST*#5483), anti-IRF3 (ET1612-14, *HUABIO*), and anti-phospho-IRF3 (p-IRF3, ET1608-22, *HUABIO*).

### Validation of protein expression by immunohistochemistry

Immunohistochemistry (IHC) were performed as previously described.^50^ PCa and normal tissues were obtained from radical prostatectomy specimens at the Urology Center, Shanghai General Hospital. Prostate tissues were fixed by immersion in 10% formalin overnight at 4 °C and subsequently stored in PBS prior to paraffin embedding. Paraffin-embedded tissue specimens were sectioned at a thickness of 4 *μ*m. Following deparaffinization with xylene and subsequent rehydration, antigen retrieval was conducted. Endogenous peroxidase activity was inhibited using 3% hydrogen peroxide in methanol. The sections were then incubated with primary antibodies targeting PCBP2 (1:200 dilution, 15070-1-AP, *Proteintech*). Additional IHC images were downloaded from the Human Protein Atlas (HPA) database. The expression levels of key genes were compared between tumor and normal tissues.

### CCK-8 assay

Cell proliferation was assessed using the Cell Counting Kit-8 (CCK-8, *New Cell & Molecular Biotech*, China). Transfected prostate cancer cells were seeded in 96-well plates (2000 cells/well) in 100 *μ*l of 1640 medium supplemented with 10% FBS and cultured for 24, 72, and 96 h. *CCK*-*8* assay was performed as previously described.^51^

### EdU assay

EdU cell proliferation staining was conducted using the EdU kit (BeyoClick™ EDU-555, China). PCa cells (2 × 10^4^ cells/well) were seeded into 12-well plates and incubated overnight at 37 °C. Subsequently, the cells were transfected with either control siRNA or siPCBP2 and allowed to stabilize for 24 h. After stabilization, the cells were incubated with EdU for 2 h, fixed with 4% paraformaldehyde for 15 min, and permeabilized with 0.3% Triton X-100 for another 15 min. The Click Reaction Mixture was then applied, and the cells were incubated for 30 min at room temperature in the dark, followed by staining with Hoechst 33 342 for 10 min.

### Transwell assay

The Transwell assay utilized 24-well chambers (*Corning Inc.*). After 48 h of transfection, 1 × 10^5^ PCa cells in 100 *μ*l serum-free medium were seeded in the upper chamber without (for migration assay) or with Matrigel placement (for invasion assay). The lower chambers contained 500 *μ*l of 10% FBS medium. After 24 h at 37 °C and 5% CO_2_, non-migrated cells were removed. The migrated and invaded cells were then fixed in 4% methanol and stained with 0.05% crystal violet, each for 15 min at room temperature, followed by microscopic imaging.

### Scratch assay

A total of 1 × 10^5^ DU145 and PC3 cells, which had been transfected with either PCBP2-siRNA or control siRNA, were seeded into six-well plates and incubated overnight. A uniform scratch was introduced into the cell monolayer using a sterile pipette tip. Subsequently, cell debris was gently removed by washing with PBS, and the cells were cultured in serum-free medium at 37 °C for 48 h. Images of the migrating cells were acquired at 0, 24, and 48 h post-scratch using an inverted microscope (Leica Microsystems GmbH, Wetzlar, Germany) at 10× magnification. Cell migration was assessed by quantifying the percentage of wound closure in five independent experiments, and the data were analyzed using ImageJ software.

### Statistical analysis

Data processing and statistical analysis were conducted using R version 4.1.1. The Wilcoxon signed-rank test evaluated differences between two groups, while the Kruskal–Wallis test assessed variations among more than two groups. Survival analyses utilized the Kaplan–Meier method and log-rank test, with univariate Cox regression analysis identifying prognostic factors. Spearman's correlation analysis was also applied. Statistical significance was determined at P < 0.05.

## SUPPLEMENTARY MATERIAL

See the supplementary material for the screening, validation, functional analysis, pan-cancer evaluation, and IHC confirmation of PCBP2 as a core cancer-intrinsic evasion gene.

## Data Availability

Complete scRNA-seq and bulk RNA-seq data are available online through the GEO portal under Project Accession Nos. GSE70769 and GSE193337. The data that support the findings of this study are available from the corresponding authors upon reasonable request.

## NOMENCLATURE

BCR: Biochemical recurrence
BCRFS: Biochemical recurrence-free survival
CCK-8: Cell counting kit-8
CIERGs: Cancer-intrinsic evasion-related genes
CNV: Copy number variation
COX: Cyclooxygenase
DEG: Differentially expressed genes
ECM: Extracellular matrix
EdU: 5-ethynyl-2′-deoxyuridine
FBS: Fetal bovine serum
GEO: Gene expression omnibus
GO: Gene ontology
GSVA: Gene set variation analysis
HPA: Human protein atlas
IHC: Immunohistochemistry
KEGG: Kyoto Encyclopedia of Genes and Genomes
KM: Kaplan–Meier
LASSO: Least absolute shrinkage and selection operator
NMF: Nonnegative matrix factorization
PCa: Prostate cancer
PCBP2: Poly(rC)-Binding Protein 2
PSMA-PET: Prostate-specific membrane antigen positron-emission tomography
qRT-PCR: Quantitative reverse transcription polymerase chain reaction
RP: Radical prostatectomy
scRNA-seq: Single-cell RNA sequencing
TCGA: The Cancer Genome Atlas
TGF-beta: Transforming growth factor-beta
TIDE: Tumor immune dysfunction and exclusion
TME: Tumor microenvironment
t-SNE: t-distributed stochastic neighbor embedding
UMAP: Uniform manifold approximation and projection
